# The genome sequence of the Adonis blue, *Lysandra bellargus *(Rottemburg, 1775)

**DOI:** 10.12688/wellcomeopenres.18330.1

**Published:** 2022-10-12

**Authors:** Konrad Lohse, Alex Hayward, Roger Vila, Wellcome Sanger Institute Tree of Life programme, Wellcome Sanger Institute Scientific Operations: DNA Pipelines collective, Tree of Life Core Informatics collective, Caitlin Howe, Darwin Tree of Life Consortium

**Affiliations:** 1 Institute of Evolutionary Biology, University of Edinburgh, Edinburgh, UK; 2 College of Life and Environmental Sciences, Department of Biosciences, University of Exeter, Exeter, UK; 3 Institut de Biologia Evolutiva, CSIC - Universitat Pompeu Fabra, Barcelona, Spain; 4 Independent researcher, Cambridge, UK

**Keywords:** Lysandra bellargus, Adonis blue, genome sequence, chromosomal, Lepidoptera

## Abstract

We present a genome assembly from an individual female *Lysandra bellargus *(the Adonis blue; Arthropoda; Insecta; Lepidoptera; Lycaenidae). The genome sequence is 529 megabases in span. The majority of the assembly (99.93%) is scaffolded into 46 chromosomal pseudomolecules with the W and Z sex chromosomes assembled. The complete mitochondrial genome was also assembled and is 15.6 kilobases in length. Gene annotation of this assembly on Ensembl has identified 13,249 protein coding genes.

## Species taxonomy

Eukaryota; Metazoa; Ecdysozoa; Arthropoda; Hexapoda; Insecta; Pterygota; Neoptera; Endopterygota; Lepidoptera; Glossata; Ditrysia; Papilionoidea; Lycaenidae; Polyommatinae; Polyommatini; Polyommatina, *Lysandra*; *Lysandra bellargus* (Rottemburg, 1775) (NCBI:txid138070).

## Background

The Adonis blue, *Lysandra bellargus* (Rottemburg, 1775), is a butterfly belonging to the gossamer-wing butterfly family Lycaenidae. It inhabits the western Palearctic region, being found most commonly in southern and central Europe, western Russia, Turkey, Transcaucasia, Caucasus, north Iraq and Iran. Its presence has been confirmed in northern Morocco and found occasionally as far north as southern Sweden ( Raper, 2021). While *L. bellargus* is listed as a species of Least Concern on the IUCN Red List of Europe ( van Swaay *et al.,* 2010), it is considered a vulnerable species in the UK ( Fox *et al.,* 2022). Within the UK, the Adonis blue is at its northern range limit and exists primarily in southern counties, such as Dorset, Wiltshire, Kent, Sussex and Surrey, as well as the Isle of Wight. Populations in the UK have been in general decline since the 1950s ( Thomas, 1983) and were severely reduced in the late 1970s after a drought caused widespread damage to the host plant ( Harper *et al.,* 2003). However, there is evidence of colonies of less than 50 individuals recovering to populations of 60,000 over five years ( Bourn *et al.,* 1998).

The Adonis blue is bivoltine, prefers calcareous grassland in sheltered, warm conditions and lays its eggs on horseshoe vetch ( *Hippocrepis comosa*). The Adonis blue is named after the sky-blue wings of the males, which are surrounded by a black rim with a white margin. The female is chocolate brown with a small number of blue scales near the base of the wings ( Still, 1996). The caterpillar is dark green with spines and has yellow stripes lining its back and sides ( Carter & Hargreaves, 1986).

The karyotype of the Adonis blue is unusual in that it has 45 chromosomes, while most butterflies in Lycaenidae have a conserved haploid chromosome number of either 23 or 24 ( Lorkovic, 1990). The genome sequence will offer further insight into lepidopteran genome evolution.

## Genome sequence report

**Figure 1.**
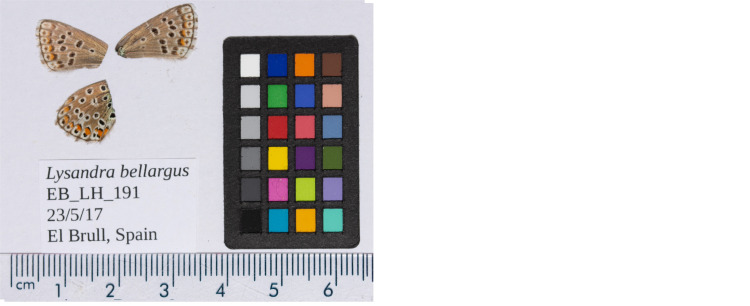
Fore and hind wings of the female *Lysandra bellargus* specimen from which the genome was sequenced. Dorsal (left) and ventral (right) surface view of wings from specimen EB_LB_191 (ilLysBell1) from El Brull, Catalunya, Spain, used to generate Pacific Biosciences, 10X genomics and Hi-C data.

The genome was sequenced from a single female *L. bellargus* collected from El Brull, Catalunya, Spain ( Figure 1). A total of 52-fold coverage of Pacific Biosciences single-molecule HiFi long reads and 65-fold coverage of 10X Genomics read clouds were generated. Primary assembly contigs were scaffolded with chromosome conformation Hi-C data. Manual assembly curation corrected 207 missing/misjoins and removed 13 haplotypic duplications, reducing the assembly size by 0.53% and the scaffold number by 47.35%, and increasing the scaffold N50 by 72.56%.

**Figure 2.**
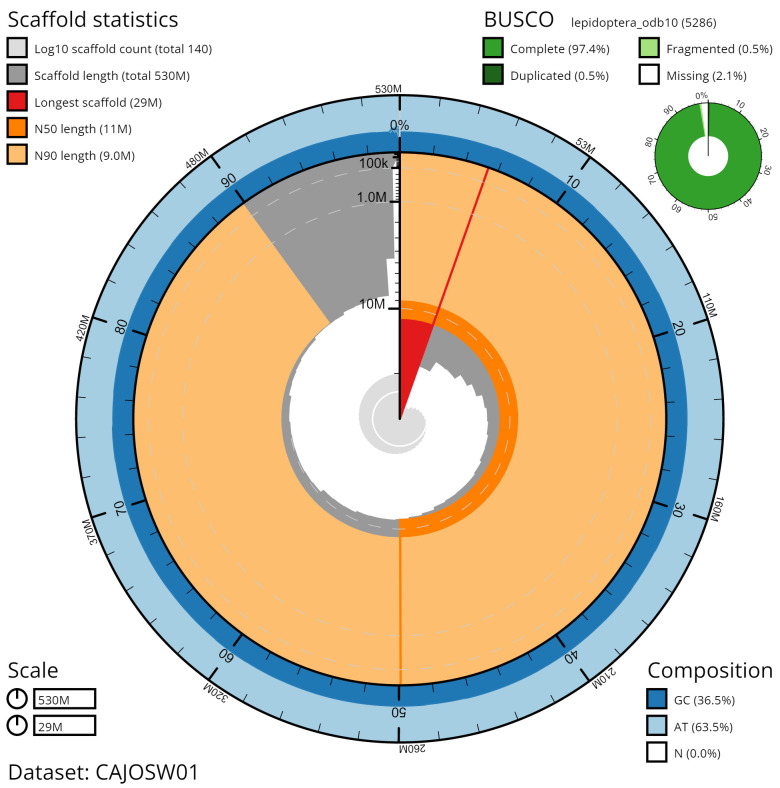
Genome assembly of *Lysandra bellargus*, ilLysBell1.1: metrics. The BlobToolKit Snailplot shows N50 metrics and BUSCO gene completeness. The main plot is divided into 1,000 size-ordered bins around the circumference with each bin representing 0.1% of the 528,933,758 bp assembly. The distribution of chromosome lengths is shown in dark grey with the plot radius scaled to the longest chromosome present in the assembly (29,060,993 bp, shown in red). Orange and pale-orange arcs show the N50 and N90 chromosome lengths (11,205,580 and 8,974,749 bp), respectively. The pale grey spiral shows the cumulative chromosome count on a log scale with white scale lines showing successive orders of magnitude. The blue and pale-blue area around the outside of the plot shows the distribution of GC, AT and N percentages in the same bins as the inner plot. A summary of complete, fragmented, duplicated and missing BUSCO genes in the lepidoptera_odb10 set is shown in the top right. An interactive version of this figure is available at https://blobtoolkit.genomehubs.org/view/ilLysBell1.1/dataset/CAJOSW01/snail.

The final assembly has a total length of 529 Mb in 139 sequence scaffolds with a scaffold N50 of 11.2 Mb ( Table 1). The majority, 99.93%, of the assembly sequence was assigned to 46 chromosomal-level scaffolds, representing 44 autosomes (numbered by sequence length) and the W and Z sex chromosomes ( Figure 2– Figure 5; Table 2). The putative W chromosome contains multiple separate regions with homology to microsporidian, insect and insect mitochondrial sequences, also displaying features consistent with its being a lepidopteran W chromosome (Hi-C interactions with lepidopteran chromosomes, telomeric repeats consistent with a lepidopteran origin, high repeat density, reduced coverage). We observed no evidence of extant infection with a microsporidian parasite. This suggests horizontal gene transfer from a microsporidian endosymbiont, which we have previously observed in other lepidopteran W chromosomes.

**Table 1.** Genome data for *Lysandra bellargus*, ilLysBell1.1.

*Project accession data*	
Assembly identifier	ilLysBell1.1
Species	*Lysandra bellargus*
Specimen	ilLysBell1 (genome assembly, Hi-C); ilLysBell2 (RNA-Seq)
NCBI taxonomy ID	138070
BioProject	PRJEB43534
BioSample ID	SAMEA7536572
Isolate information	Female, whole organism (ilLysBell1); male, whole organism (ilLysBell2)
*Raw data accessions*	
PacificBiosciences SEQUEL II	ERR6576322
10X Genomics Illumina	ERR6054513-ERR6054516
Hi-C Illumina	ERR6054517
PolyA RNA-Seq Illumina	ERR6363262
*Genome assembly*	
Assembly accession	GCA_905333045.1
*Accession of alternate haplotype*	GCA_905332955.1
Span (Mb)	529
Number of contigs	361
Contig N50 length (Mb)	2.9
Number of scaffolds	139
Scaffold N50 length (Mb)	11.2
Longest scaffold (Mb)	18.03
BUSCO * genome score	C:97.4%[S:96.9%,D:0.5%],F:0.5%,M:2.1%,n:5,286
*Genome annotation*	
Number of protein-coding genes	13,249
Average length of coding sequence (bp)	1,443.19
Average number of exons per transcript	7.00
Average intron size (bp)	2,238.35

*BUSCO scores based on the lepidoptera_odb10 BUSCO set using v5.3.2. C= complete [S= single copy, D=duplicated], F=fragmented, M=missing, n=number of orthologues in comparison. A full set of BUSCO scores is available at https://blobtoolkit.genomehubs.org/view/ilLysBell1.1/dataset/CAJOSW01/busco.

**Figure 3.**
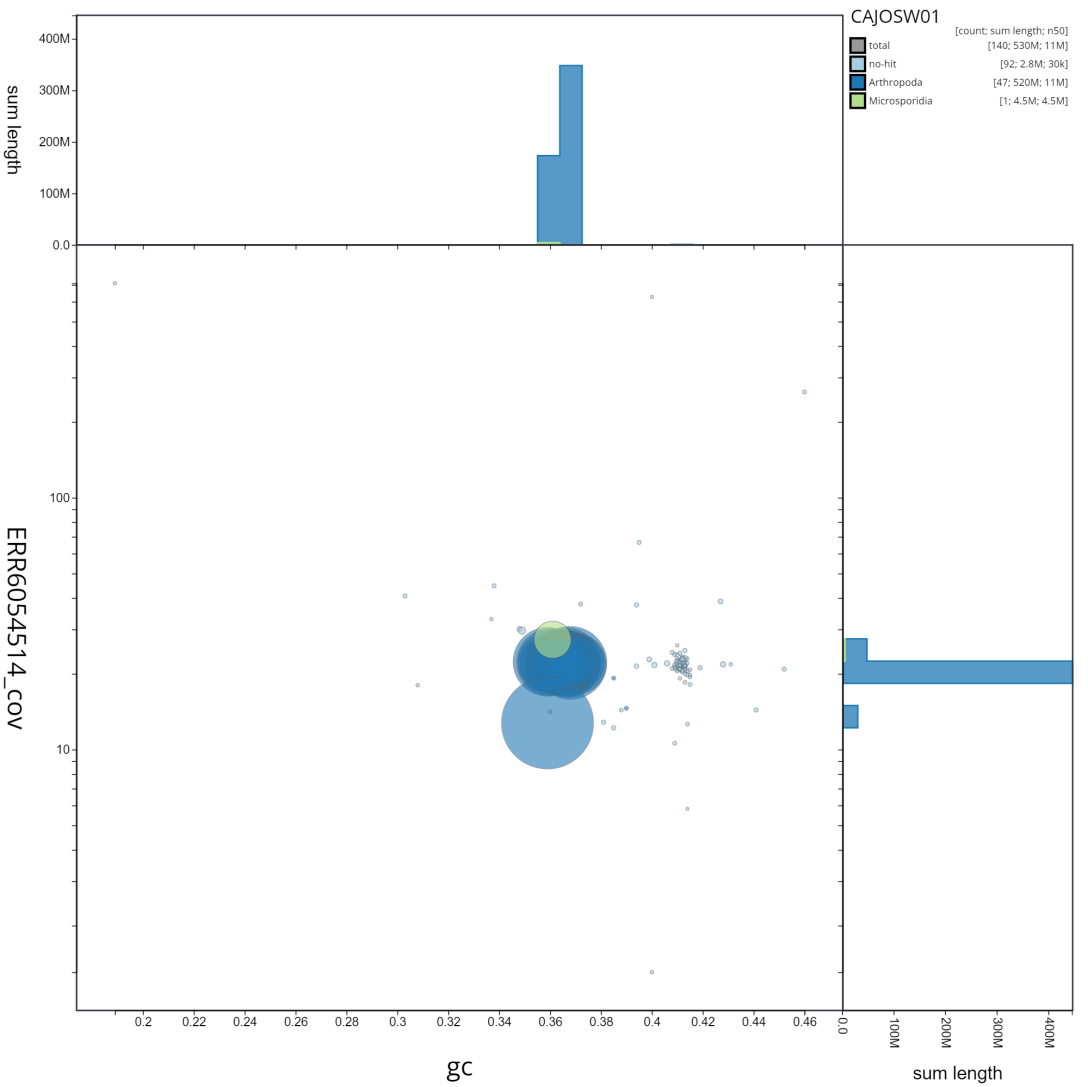
Genome assembly of *Lysandra bellargus*, ilLysBell1.1: GC coverage. BlobToolKit GC-coverage plot. Scaffolds are coloured by phylum. Circles are sized in proportion to scaffold length. Histograms show the distribution of scaffold length sum along each axis. An interactive version of this figure is available at https://blobtoolkit.genomehubs.org/view/ilLysBell1.1/dataset/CAJOSW01/blob.

**Figure 4.**
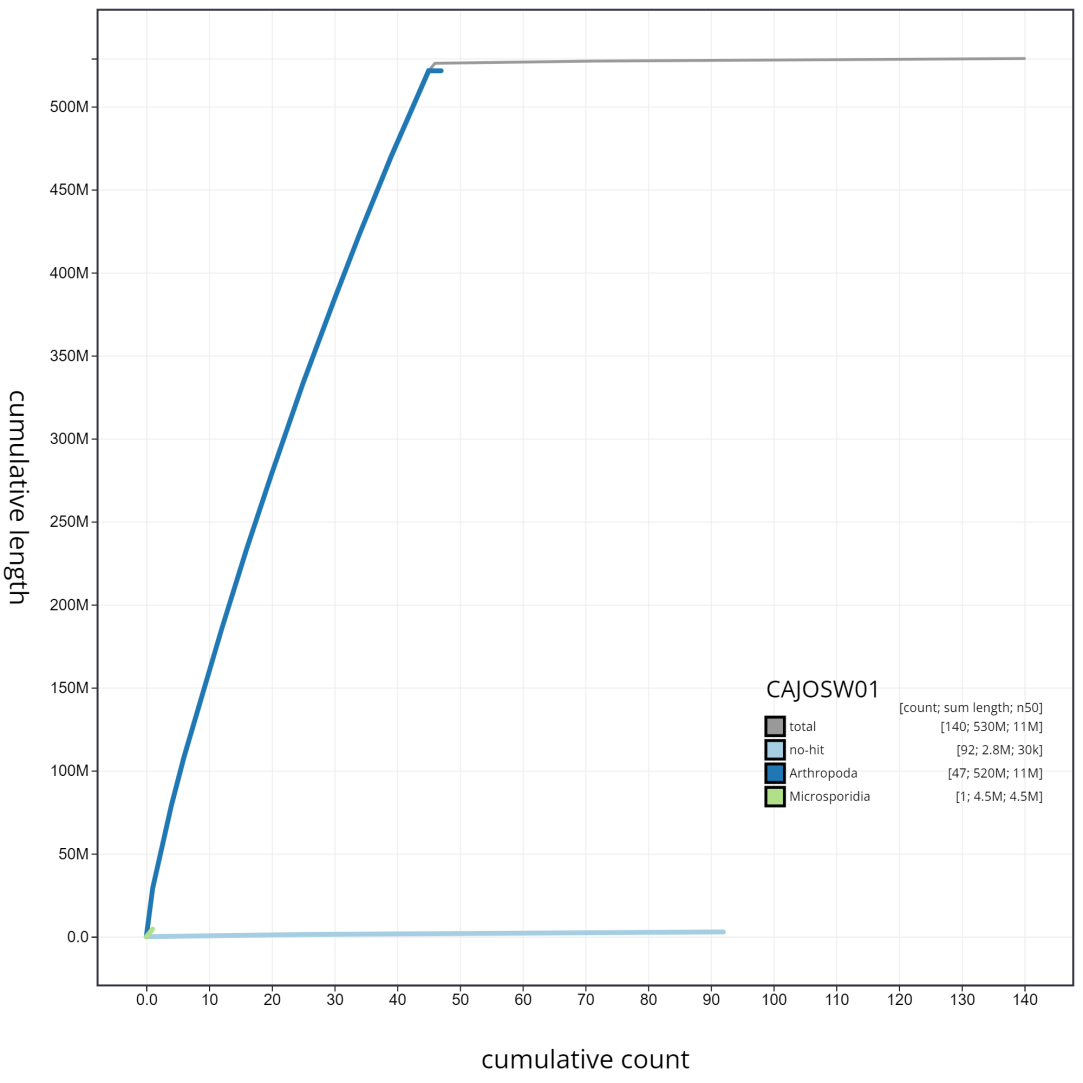
Genome assembly of *Lysandra bellargus*, ilLysBell1.1: cumulative sequence. BlobToolKit cumulative sequence plot. The grey line shows cumulative length for all scaffolds. Coloured lines show cumulative lengths of scaffolds assigned to each phylum using the buscogenes taxrule. An interactive version of this figure is available at https://blobtoolkit.genomehubs.org/view/ilLysBell1.1/dataset/CAJOSW01/cumulative.

**Figure 5.**
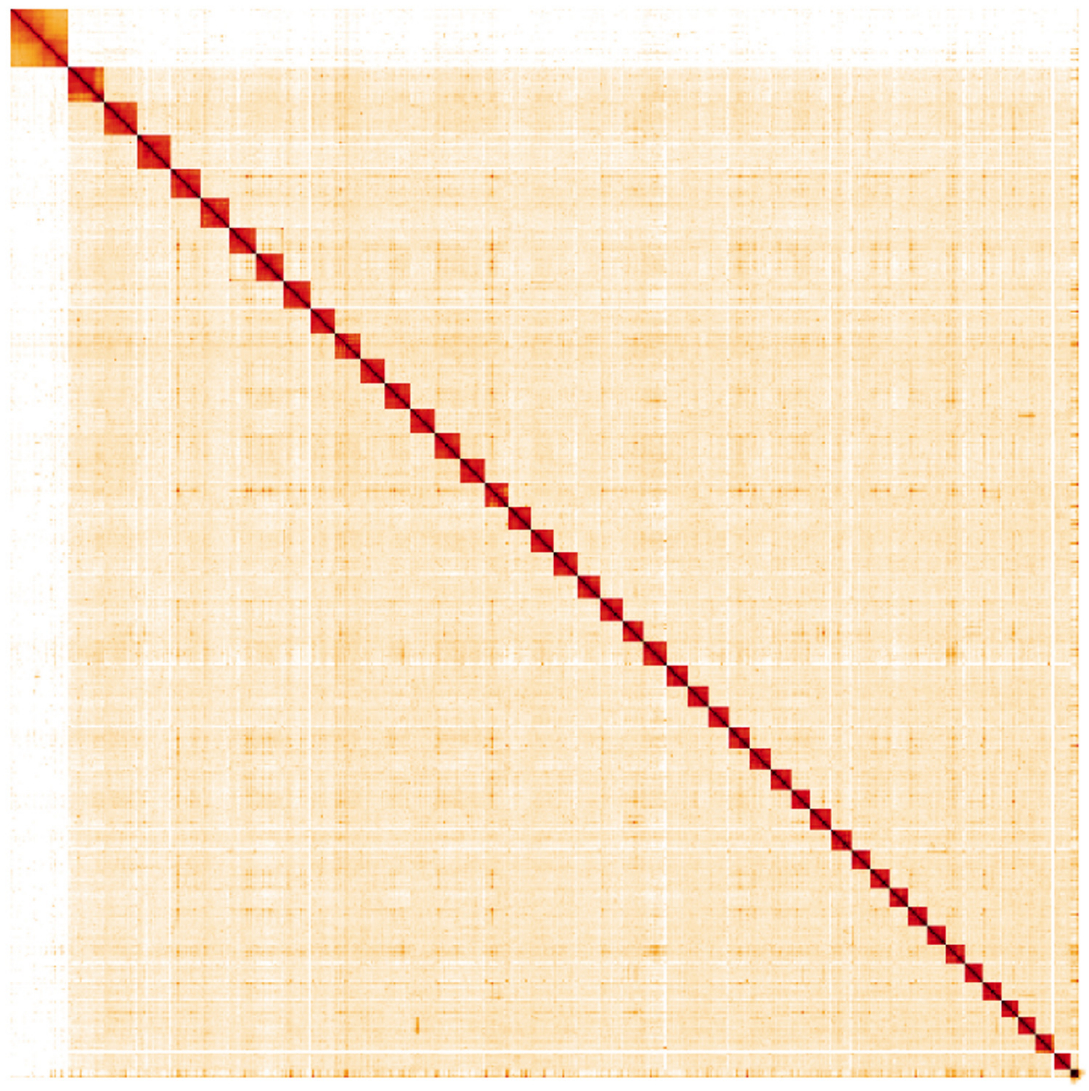
Genome assembly of *Lysandra bellargus*, ilLysBell1.1: Hi-C contact map. Hi-C contact map of the ilLysBell1.1 assembly, visualised in HiGlass. Chromosomes are arranged in size order from left to right and top to bottom. The interactive Hi-C map can be viewed at https://genome-note-higlass.tol.sanger.ac.uk/l/?d=BZpgv8y6Q_2udwxedQCuYA.

**Table 2.** Chromosomal pseudomolecules in the genome assembly of *Lysandra bellargus*, ilLysBell1.1.

INSDC accession	Chromosome	Size (Mb)	GC%
HG995320.1	1	18.03	36.8
HG995321.1	2	16.11	36.6
HG995322.1	3	16	35.9
HG995323.1	4	14.92	36
HG995324.1	5	14.16	36.7
HG995325.1	6	13.29	36.5
HG995326.1	7	13.03	36.4
HG995327.1	8	12.89	36.3
HG995328.1	9	12.69	36.7
HG995329.1	10	12.63	37
HG995330.1	11	12.36	36.2
HG995331.1	12	12.19	36.7
HG995332.1	13	12.19	36.4
HG995333.1	14	12.14	36.5
HG995334.1	15	12.01	36.7
HG995335.1	16	11.66	36.6
HG995336.1	17	11.44	35.9
HG995337.1	18	11.21	36.6
HG995338.1	19	11.11	36.5
HG995339.1	20	11.09	36.5
HG995340.1	21	11.03	36.4
HG995341.1	22	10.81	36.6
HG995342.1	23	10.76	37
HG995343.1	24	10.71	36.4
HG995344.1	25	10.24	36.2
HG995345.1	26	10.16	36.5
HG995346.1	27	10.09	36.2
HG995347.1	28	10.09	36.4
HG995348.1	29	10.04	36.3
HG995349.1	30	9.99	37
HG995350.1	31	9.86	35.9
HG995351.1	32	9.83	36.4
HG995352.1	33	9.52	36.6
HG995353.1	34	9.46	36.2
HG995354.1	35	9.37	36.2
HG995355.1	36	9.26	36.1
HG995356.1	37	9.25	36.7
HG995357.1	38	8.99	36.7
HG995358.1	39	8.97	36.5
HG995359.1	40	8.94	36.6
HG995360.1	41	8.65	36.4
HG995361.1	42	8.59	36.5
HG995362.1	43	8.41	36.3
HG995363.1	44	8.29	36.8
HG995364.1	W	4.54	36.1
HG995319.1	Z	29.06	35.9
HG995365.1	MT	0.02	19.1
-	Unplaced	2.86	40.3

The assembly has a BUSCO v5.3.2 ( Manni *et al.,* 2021) completeness of 97.4% (single 96.9%, duplicated 0.5%) using the lepidoptera_odb10 reference set (n=5,286). While not fully phased, the assembly deposited is of a single haplotype. Contigs corresponding to the second haplotype have also been deposited.

## Genome annotation report

The ilLysBell1.1 genome was annotated using the Ensembl rapid annotation pipeline ( Table 1; Lysandra bellargus Ensembl page). The resulting annotation includes 24,348 transcribed mRNAs from 13,249 protein-coding and 2,895 non-coding genes. There is an average of 7.00 exons and 6.00 introns per canonical protein coding transcript, with an average intron length of 2,238.35 bases.

## Methods

### Sample acquisition and nucleic acid extraction

Two *L. bellargus* specimens (ilLysBell1, female; ilLysBell2, male) were collected using a net from El Brull, Catalunya, Spain (latitude 41.8103, longitude 2.3054) by Konrad Lohse (University of Edinburgh) and Alex Hayward (University of Exeter). The specimens were identified by Roger Vila (Institut de Biologia Evolutiva, Barcelona) and flash frozen from live in a dry shipper. 

DNA was extracted at the Scientific Operations Core, Wellcome Sanger Institute. The ilLysBell1 sample was weighed and dissected on dry ice with head tissue set aside for Hi-C sequencing. Whole organism tissue was disrupted by manual grinding in lysis buffer with a disposable pestle. Fragment size analysis of 0.01–0.5 ng of DNA was then performed using an Agilent FemtoPulse. High molecular weight (HMW) DNA was extracted using the Qiagen MagAttract HMW DNA extraction kit. Low molecular weight DNA was removed from a 200-ng aliquot of extracted DNA using 0.8X AMpure XP purification kit prior to 10X Chromium sequencing; a minimum of 50 ng DNA was submitted for 10X sequencing. HMW DNA was sheared into an average fragment size between 12–20 kb in a Megaruptor 3 system with speed setting 30. Sheared DNA was purified by solid-phase reversible immobilisation using AMPure PB beads with a 1.8X ratio of beads to sample to remove the shorter fragments and concentrate the DNA sample. The concentration of the sheared and purified DNA was assessed using a Nanodrop spectrophotometer and Qubit Fluorometer and Qubit dsDNA High Sensitivity Assay kit. Fragment size distribution was evaluated by running the sample on the FemtoPulse system.

RNA was extracted from the whole organism tissue of ilLysBell2 in the Tree of Life Laboratory at the WSI using TRIzol, according to the manufacturer’s instructions. RNA was then eluted in 50 μl RNAse-free water and its concentration assessed using a Nanodrop spectrophotometer and Qubit Fluorometer using the Qubit RNA Broad-Range (BR) Assay kit. Analysis of the integrity of the RNA was done using Agilent RNA 6000 Pico Kit and Eukaryotic Total RNA assay.

### Sequencing

Pacific Biosciences HiFi circular consensus and 10X Genomics Chromium read cloud sequencing libraries were constructed according to the manufacturers’ instructions. Sequencing was performed by the Scientific Operations core at the Wellcome Sanger Institute on Pacific Biosciences SEQUEL II (HiFi), Illumina HiSeq X Ten and Illumina HiSeq 4000 (RNA-Seq) instruments. Hi-C data were generated in the Tree of Life laboratory from remaining whole organism tissue of ilLysBell1 using the Arima v2 kit and sequenced on a NovaSeq 6000 instrument.

### Genome assembly

Assembly was carried out with Hicanu ( Nurk *et al.,* 2020); haplotypic duplication was identified and removed with purge_dups ( Guan *et al.,* 2020). One round of polishing was performed by aligning 10X Genomics read data to the assembly with longranger align, calling variants with freebayes ( Garrison & Marth, 2012). The assembly was then scaffolded with Hi-C data ( Rao *et al.,* 2014) using SALSA2 ( Ghurye *et al.,* 2019). The assembly was checked for contamination and corrected using the gEVAL system ( Chow *et al.,* 2016) as described previously ( Howe *et al.,* 2021). Manual curation ( Howe *et al.,* 2021) was performed using gEVAL, HiGlass ( Kerpedjiev *et al.,* 2018) and Pretext. The mitochondrial genome was assembled using MitoHiFi ( Uliano-Silva *et al.,* 2021), which performs annotation using MitoFinder ( Allio *et al.,* 2020). The genome was analysed and BUSCO scores generated within the BlobToolKit environment ( Challis *et al.,* 2020). Table 3 contains a list of all software tool versions used, where appropriate.

**Table 3.** Software tools used.

Software tool	Version	Source
Hicanu	2.1	Nurk *et al.,* 2020
purge_dups	1.2.3	Guan *et al.,* 2020
SALSA2	2.2	Ghurye *et al.,* 2019
longranger align	2.2.2	https://support.10xgenomics. com/genome-exome/software/ pipelines/latest/advanced/ other-pipelines
freebayes	1.3.1-17- gaa2ace8	Garrison & Marth, 2012
MitoHiFi	1.0	Uliano-Silva *et al.,* 2021
HiGlass	1.11.6	Kerpedjiev *et al.,* 2018
PretextView	0.2.x	https://github.com/wtsi-hpag/ PretextView
BlobToolKit	3.2.6	Challis *et al.,* 2020

### Genome annotation

The Ensembl gene annotation system ( Aken *et al.,* 2016) was used to generate annotation for the *Lysandra bellargus* assembly (GCA_905333045.1). Annotation was created primarily through alignment of transcriptomic data to the genome, with gap filling via protein-to-genome alignments of a select set of proteins from UniProt ( UniProt Consortium, 2019).

## Data Availability

European Nucleotide Archive: Lysandra bellargus (Adonis blue). Accession number PRJEB43534; https://identifiers.org/ena.embl/PRJEB43534 The genome sequence is released openly for reuse. The *L. bellargus* genome sequencing initiative is part of the Darwin Tree of Life (DToL) project. All raw sequence data and the assembly have been deposited in INSDC databases. Raw data and assembly accession identifiers are reported in Table 1.
