# Supplementary material for: The genome sequence of the Adonis blue, Lysandra bellargus (Rottemburg, 1775)
Source: Wellcome Open Res. 2022 Oct 12;7:255. [Version 1] doi: 10.12688/wellcomeopenres.18330.1 (PMC10090861; doi:10.12688/wellcomeopenres.18330.1)
Supplement: Peer review history

# Peer review: Reviewer response for version 1

**DOI:** 10.21956/wellcomeopenres.20317.r56068

**Published:** 2023-04-24

**Authors:** Maria de la Paz Celorio-Mancera

**Affiliations:** 1 Stockholm University, Stockholm, Sweden

The authors present the genome assembly for the Adonis blue, *Lysandra bellargus*. The species is of interest since it is considered vulnerable in the UK and because of two other observations: its potential capacity of population recovery from a limited number of individuals and its unusual number of chromosomes when compared to that observed among the family Lycaenidae. In general, the article reports a well conducted effort to obtain the genome of this species of butterfly. My concerns are related to the rationale and the protocols used for the study.

 First, if the species is of interest due to its chromosome number, would the authors have observed or expect intraspecific variability in the karyotype of the species? This exceptional although possible circumstance is not addressed. Unfortunately, since the study does not provide a chromosome level genome (no linkage mapping done), the authors can not conclude the actual number of chromosomes found in their specimen.

 Second, there is a general effort in the community to provide genomes that are as homozygous as possible. I understand that inbreeding and laboratory breeding is difficult in some species. However, I miss the effort from the authors to provide a somewhat homozygous genome and therefore nucleotide diversity as a potential concern if using this genome as a reference.

 I found the BlobToolKit a very nice tool for the visualisation of genome data.

Are sufficient details of methods and materials provided to allow replication by others?

Yes

Is the rationale for creating the dataset(s) clearly described?

Partly

Are the datasets clearly presented in a useable and accessible format?

Yes

Are the protocols appropriate and is the work technically sound?

Partly

Reviewer Expertise:

Plant Biology, Plant-Insect Interactions, Enzymology, Proteomics, Transcriptomics, Population Genomics

I confirm that I have read this submission and believe that I have an appropriate level of expertise to confirm that it is of an acceptable scientific standard, however I have significant reservations, as outlined above.

# Peer review: Reviewer response for version 1

**DOI:** 10.21956/wellcomeopenres.20317.r55785

**Published:** 2023-04-14

**Authors:** Pasi Rastas

**Affiliations:** 1 Institute of Biotechnology, University of Helsinki, Helsinki, Helsinki, Finland

The authors present a chromosome level assembly of the Adonis blue -butterfly using Pacbio Hifi, Hi-C and 10x data. The assembly is of high quality.

 The assembly pipeline is described very briefly and is a bit confusing. Here are some questions about the pipeline and the article:

- Were the default parameters used in the software?
- Were the software applied in this order: Hicanu, purge_dups, polishing, SALSA2, manual curation?
- I am wondering, this as the genome sequence report says: "Manual assembly curation corrected 207 missing/misjoins and removed 13 haplotypic duplications,..." - Is the manual curation here referring the manual curation of gEval, HiGlass and Pretext? Or are the haplotypes removed by purge_dups earlier? 
- One of the main concerns is the used software purge_dups. Based on its manual, it removes haplotypes only from the contig ends (on default parameters). But when assembly errors are corrected (with Hi-C), new contig ends are created and these could contain haplotypic duplications.
- There could be more info (and citation) about other lepidopteran W chromosomes. Typically the chromosome synteny in lepidoptera is high, maybe an alignment to some other species could be informative (chromosomes Z, W and/or autosomes)?
- Maybe it could be stated more clearly what reduced coverage means for W (and Z) chromosomes. Are the differences shown in the Figure 3? 
- How much did the 10x data polishing improve the genome? Did the Hifi reads / assembled contigs have worse base qualities than obtained by Illumina sequencing? Are the 10x UMIs used in the process? They could be used for scaffolding and phasing? 
- There is no mention of the use of RNAseq data.

Are sufficient details of methods and materials provided to allow replication by others?

Partly

Is the rationale for creating the dataset(s) clearly described?

Yes

Are the datasets clearly presented in a useable and accessible format?

Yes

Are the protocols appropriate and is the work technically sound?

Partly

Reviewer Expertise:

Genome assemby, population genetics

I confirm that I have read this submission and believe that I have an appropriate level of expertise to confirm that it is of an acceptable scientific standard, however I have significant reservations, as outlined above.

# Peer review: Reviewer response for version 1

**DOI:** 10.21956/wellcomeopenres.20317.r56069

**Published:** 2023-04-11

**Authors:** Benoit Nabholz

**Affiliations:** 1 Université Montpellier, Institut Universitaire de France, Montpellier, France

The authors presents the chromosome scale assembly of the Adonis blue, *Lysandra bellargu* *s.*

 The genome is of high quality like the other genomes produced by the Darwin Tree of Life project. All the links and the datasets presented are publicly available.

 The authors indicate that W chromosomes contains sequences homologous to microsporidian and suggest that this is likely a horizontal transfer. The authors indicate that this “...have previously observed in other lepidopteran W chromosomes” but without any reference to this claims. The authors should provide a reference to this claim.

 Overall, I would like to thank the Darwin Tree of Life Consortium for the whole project. It provides high quality genomes that are quickly and transparently available. This project is really well organised and its very valuable to the scientific community.

Are sufficient details of methods and materials provided to allow replication by others?

Yes

Is the rationale for creating the dataset(s) clearly described?

Yes

Are the datasets clearly presented in a useable and accessible format?

Yes

Are the protocols appropriate and is the work technically sound?

Yes

Reviewer Expertise:

Population genomics. Molecular evolution. Ornithology and entomology.

I confirm that I have read this submission and believe that I have an appropriate level of expertise to confirm that it is of an acceptable scientific standard.

# Peer review: Reviewer response for version 1

**DOI:** 10.21956/wellcomeopenres.20317.r55132

**Published:** 2023-03-17

**Authors:** Ole Kristian Tørresen

**Affiliations:** 1 Centre for Ecological and Evolutionary Synthesis, University of Oslo, Oslo, Norway

The authors have assembled a genome of the Adonis blue, *Lysandra bellargus *(Rottemburg, 1775), using a combination of PacBio HiFi, 10X Genomics and Arima Hi-C reads. The metrics with regards to N50 contig and scaffold, BUSCO genes and number of sequences in chromosomes, are all very good.

 The methods used are all standard and expected to give good results. However, it is not given enough details to replicate the process. For instance, were all the software run with default options, or were any parameters adjusted?

 How much did the single round of polishing affect the assembly? Considering that the HiFi data is of quite high quality from before. How the polishing affected the assembly could assessed by quality values and k-mer completeness as reported by Merqury, seeing how this varies between different assemblies. It would at least have been nice to see the values for the final assembly.

Are sufficient details of methods and materials provided to allow replication by others?

Partly

Is the rationale for creating the dataset(s) clearly described?

Yes

Are the datasets clearly presented in a useable and accessible format?

Yes

Are the protocols appropriate and is the work technically sound?

Yes

Reviewer Expertise:

Genomics

I confirm that I have read this submission and believe that I have an appropriate level of expertise to confirm that it is of an acceptable scientific standard.
